# Design and rationale of the WE-CARE-HF-CMR trial: Cardiorenal care on wheels for asymptomatic heart failure patients (NCT07185100)

**DOI:** 10.1016/j.jocmr.2026.102745

**Published:** 2026-05-15

**Authors:** Jeffrey Ji-Peng Li, Gisela Thiede, Collin Götze, Vasileios Exarchos, Cyrill Meuwly, Stefanie Maria Werhahn, Rebecca Elisabeth Beyer, Maximilian Leo Müller, Tamar Bigvava, Christian Stehning, Bjoern Andrew Remppis, Johannes Wieditz, Stefan Sack, Tobias Heer, Michael Sroka, Eike Nagel, Allison G. Hays, Grietje Duvigneau, Stefan Blankenberg, Henning Steen, Norbert Frey, Stephan Baldus, Katharina Seuthe, Rolf Lorbach, Florian Rödicker, Sarah Scharf, Ingo Hilgendorf, Ulf Landmesser, Tim Friede, Volkmar Falk, Patrick Doeblin, Sebastian Kelle

**Affiliations:** a Department of Cardiology, Angiology, and Intensive Care Medicine, Berlin, Germany; b Charité – Universitätsmedizin Berlin, corporate member of Freie Universität Berlin and Humboldt-Universität zu Berlin, Berlin, Germany; c German Centre for Cardiovascular Research (DZHK), Partner Site Berlin, Germany; d Department of Cardiology, Angiology, and Intensive Care Medicine, Berlin, Germany; e Department of Cardiology, Angiology, and Intensive Care Medicine, Berlin, Germany; f Philips Healthcare, Clinical Science, Hamburg, Germany; g Heart and Vascular Center Bad Bevensen, Bad Bevensen, Germany; h Department of Medical Statistics, University Medical Center Göttingen, Göttingen, Germany; i Department of Cardiology, München Klinik Neuperlach, Academic Teaching Hospital of LMU Klinikum, Munich, Germany; j Fraport AG, Medical Services, Frankfurt, Germany; k Institute for Experimental and Translational Cardiovascular Imaging, Goethe University Frankfurt am Main, Frankfurt, Germany; l German Centre for Cardiovascular Research (DZHK), Partner Site Rhine-Main, Frankfurt, Germany; m Division of Cardiology, Department of Medicine, Johns Hopkins University School of Medicine, Baltimore, Maryland, USA; n Praxis Dres. med. Rutsch&Duvigneau GbR, Hamburg, Germany; o Philips GmbH, Company Doctor, Hamburg, Germany; p University Heart and Vascular Center Hamburg, University Medical Center Hamburg-Eppendorf, Hamburg, Germany; q German Centre for Cardiovascular Research (DZHK), Partner Site Hamburg-Kiel-Luebeck, Hamburg, Germany; r Center for Population Health Innovation, Hamburg, Germany; s Cardio-CARE, Davos, Switzerland; t Department of Cardiology, Angiology and Pneumology, Heidelberg University, Germany; u German Centre for Cardiovascular Research (DZHK), Partner Site Heidelberg/Mannheim, Germany; v Department III of Internal Medicine, Heart Center, Faculty of Medicine and University Hospital Cologne, University of Cologne, Germany; w Center for Molecular Medicine Cologne, University of Cologne, Germany; x Stadtwerke Köln GmbH, Company Doctor, Cologne, Germany; y AstraZeneca, Medical Affairs, Hamburg, Germany; z German Centre for Cardiovascular Research (DZHK), partner site Lower Saxony, Göttingen, Germany; aa Department of Cardiothoracic and Vascular Surgery, Deutsches Herzzentrum der Charité, Berlin, Germany

**Keywords:** Stage B heart failure, Pre-heart failure, Screening, Prevention, Cardiac magnetic resonance imaging, Observational trial

## Abstract

**Background:**

Heart failure (HF) is a significant public health concern. Early detection, particularly during the asymptomatic stage, is essential for prompt intervention and preventing progression. Conventional measures, such as left ventricular ejection fraction (LVEF) have limited sensitivity. However, left ventricular global longitudinal strain (GLS) can detect subclinical myocardial dysfunction. Preliminary data from the previous HERZCHECK study showed that 23% (1023/4509) of participants in rural Germany had GLS-defined subclinical pre-HF (stage B HF) when examined with mobile cardiac MRI units. Yet, the prevalence of asymptomatic HF in urban populations remains unclear. The WE-CARE-HF-CMR trial aims to address this issue.

**Study design and methodology:**

The WE-CARE-HF-CMR trial is a single-center prospective observational study with a cross-sectional baseline assessment and an exploratory longitudinal follow-up conducted in five urban German cities (Berlin, Cologne, Frankfurt, Hamburg, Munich; NCT07185100). Asymptomatic patients between the ages of 40 and 69 with cardiovascular risk factors but without a history of symptomatic HF are enrolled via outpatient physician referral or self-referral. Participants undergo a standardized diagnostic evaluation including a medical history review, laboratory testing, cardiovascular magnetic resonance (CMR) imaging with GLS analysis, and quality-of-life questionnaires. CMR examinations are performed using both stationary and mobile magnetic resonance imaging (MRI) units. Subclinical pre-HF (stage B HF) is defined as GLS ≥ −15%. The primary endpoint is the prevalence of subclinical pre-HF (stage B HF) in the urban population. Secondary endpoints assess therapy adherence, quality of life, and the prevalence of chronic kidney disease. CMR images are analyzed centrally following standardized protocols to ensure comparability with HERZCHECK rural data.

**Conclusion:**

The WE-CARE-HF-CMR trial will provide critical data on the prevalence of subclinical pre-HF (stage B HF) in urban populations. Using CMR-based GLS analysis with stationary and mobile units, and building on the HERZCHECK trial methodology, the study will close the diagnostic gap between rural and urban areas. These findings may ultimately support the implementation of targeted screening programs for subclinical pre-HF (stage B HF) to enable early intervention and prevent the progression to symptomatic HF.

**ClinicalTrials.gov Identifier:**

NCT07185100

## 1 Background

Heart failure (HF) is a significant public health concern worldwide. In Germany, the prevalence of clinically overt HF is greater than 30 cases per 1000 people, resulting in an associated annual healthcare cost of €25,532 per patient [1], [2]. Due to the increase in life expectancy and the higher incidence of HF among elderly people, many more individuals are at risk of developing HF in the future [3]. Individuals with established cardiovascular risk factors are particularly at risk. For example, obesity and elevated blood pressure have been linked to a ∼45% relative increase in the residual lifetime risk for HF [3]. Therefore, it is essential to detect the early asymptomatic phase of HF, particularly within this high-risk group.

Subclinical pre-HF, or stage B HF, is defined as the presence of structural or functional cardiac abnormalities without symptoms [4]. Individuals with subclinical pre-HF (stage B HF) are at an increased risk of progressing to clinically manifest (i.e., symptomatic) HF [5]. However, conventional parameters such as left ventricular ejection fraction (LVEF) have limited ability to detect early myocardial dysfunction in asymptomatic individuals [6]. Left ventricular global longitudinal strain (GLS) describes the change in myocardial length from end-diastole to end-systole in the longitudinal plane and can be assessed by echocardiography or CMR. It has emerged as a promising parameter for detecting subclinical myocardial impairment without LVEF impairment [7], [8].

The recently completed HERZCHECK study (NCT05122793) screened the population of German rural regions for early subclinical pre-HF (stage B HF) using mobile cardiac magnetic resonance imaging (MRI) units [9]. These units were centrally coordinated and telemedically supervised by an expert core center. HERZCHECK was the first initiative to collect validated, multimodal data, including imaging, biospecimens, and patient-reported outcomes, from a large enough cohort of 4509 patients in medically underserved rural areas. Preliminary data from this cohort indicates a 23% (1023/4509) prevalence of subclinical pre-HF (stage B HF) and the study successfully demonstrated the feasibility of large-scale mobile cardiac magnetic resonance (CMR) screening [10].

Although symptomatic HF is known to be more prevalent in rural areas, it is unclear whether the prevalence of subclinical pre-HF (stage B HF) is comparable between urban and rural populations with equivalent risk profiles. This is due to differences in lifestyle, environmental exposure, and infrastructure [11]. Access to healthcare is more limited and income levels are lower in rural regions compared to urban areas, whereas the urban population is exposed to higher levels of air and noise pollution [12]. Therefore, it is necessary to extend the established HERZCHECK trial methodology to an urban setting.

The WE-CARE-HF-CMR study, built on the HERZCHECK trial, was designed to assess the prevalence of subclinical pre-HF (stage B HF) in urban populations using a standardized CMR-based approach that includes both stationary and mobile imaging units.

## 2 Study design and methodology

### 2.1 Overview of study design

**Fig. 1:**
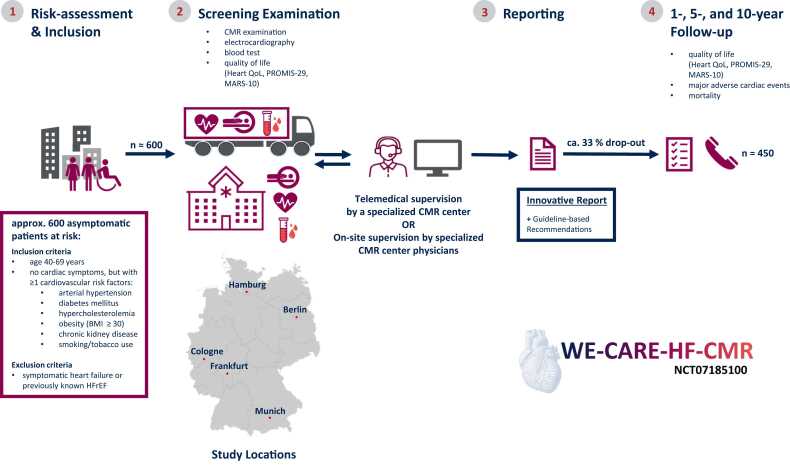
Overview of the WE-CARE-HF-CMR study design, including eligibility criteria, study locations, and study examination. *CMR* cardiovascular magnetic resonance

The WE-CARE-HF-CMR study is a single-center prospective observational study with a cross-sectional baseline assessment and an exploratory longitudinal follow-up. The objective is to determine the prevalence of subclinical pre-HF (stage B HF) in urban areas of Germany. Data will be collected in five major cities (Berlin, Cologne, Frankfurt, Hamburg, and Munich), each with a population of at least one million, using stationary and mobile cardiac MRI (Fig. 1). The results will be benchmarked against the rural prevalence data obtained from the HERZCHECK study [9].

### 2.2 Ethics

The WE-CARE-HF-CMR trial has been approved by the Ethics Committee of Charité – Universitätsmedizin Berlin in Germany (application number EA4/166/25) and has been registered with ClinicalTrials.gov (NCT07185100). The study is conducted in accordance with the Declaration of Helsinki and Good Clinical Practice guidelines. Written informed consent will be obtained from all participants.

### 2.3 Study population, screening, and enrollment

The WE-CARE-HF-CMR study enrolls asymptomatic individuals who reside in urban areas and have one or more of the following risk factors, but no known heart failure: hypercholesterolemia, arterial hypertension, obesity, smoking/tobacco consumption, chronic diabetes mellitus, and/or chronic kidney disease (CKD). The inclusion and exclusion criteria are largely identical to those of the previous study, except for the following points: for this study, individuals do not need to have existing health insurance or access to a smartphone [9]. Participants are referred by outpatient and occupational physicians, or can enroll themselves. All participants are screened for eligibility by a physician on the day of the examination.

### 2.4 Study examinations

All participants receive a standardized diagnostic work-up identical to that of the HERZCHECK trial, including medical history, laboratory testing, CMR, and quality of life (QoL) questionnaires [9].

#### 2.4.1 Medical history

The on-site physicians of the study center or remote telemedicine providers collect a focused medical history of the participants using the same questionnaire as in the HERZCHECK trial [9].

#### 2.4.2 Laboratory testing

A venous blood draw is performed, collecting a total of two ethylenediaminetetraacetic acid tubes and one serum tube of blood. Participants also submit a urine sample to examine the urinary albumin creatinine ratio (UACR). All blood and urine samples are analyzed in the Deutsches Herzzentrum der Charité laboratory at the Campus Virchow Klinikum in Berlin, Germany. The examined laboratory parameters match those of the HERZCHECK trial protocol, except that C-reactive protein was added to the panel, and electrolytes were removed [9].

#### 2.4.3 Quality of life questionnaires

The “HeartQoL” and the “PROMIS®-29 Profile” questionnaires, adopted from the HERZCHECK trial, are used to assess quality of life. To assess therapy adherence, the “MARS-10” questionnaire was also added [9]. Participants complete the questionnaires by themselves.

#### 2.4.4 Cardiovascular magnetic resonance imaging and image analysis

**Fig. 2:**
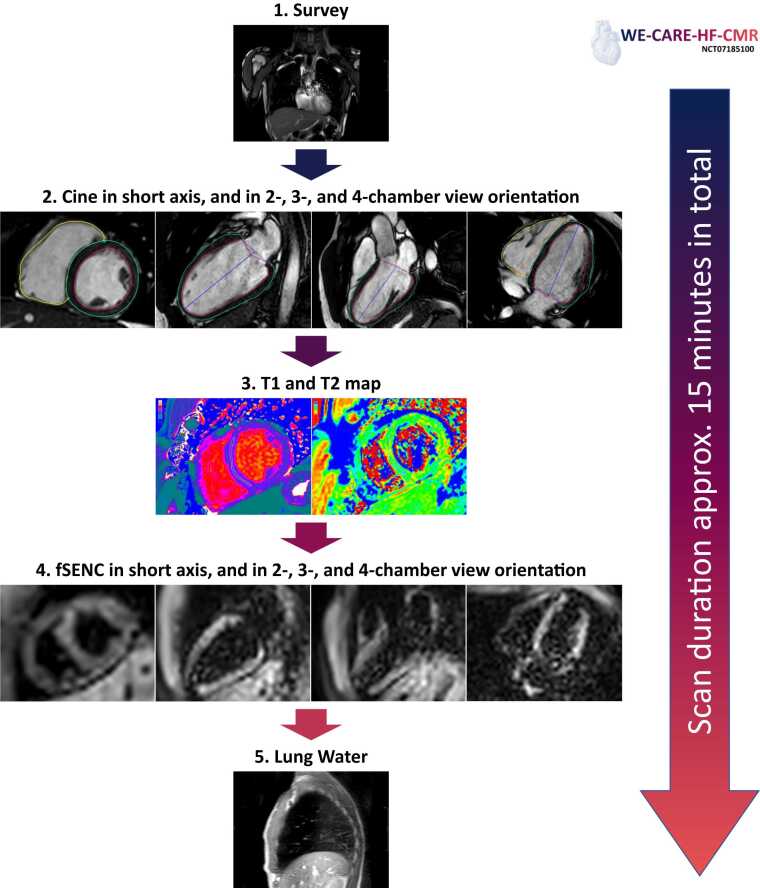
Overview of the CMR protocol used in the WE-CARE-HF-CMR trial. Similar to the previous HERZCHECK trial, no contrast- or stress-agents are administered, and it includes a survey, cine imaging, native T1 and T2 mapping. Newly added components are fSENC sequences in short- and long-axis views as well as lung water imaging sequences. *CMR* cardiac magnetic resonance, *fSENC* fast strain-encoded imaging

The contrast-free CMR protocol and image acquisition procedure are adopted from the HERZCHECK trial. The central imaging parameter used to assess cardiac function is still the left ventricular GLS, which is quantified using a feature-tracking approach [9]. Newly added components include the fast strain-encoded imaging (fSENC) sequences in long- and short-axis views, and a lung water imaging sequence (Fig. 2) [13], [14], [15]. Mapping data are not used for diagnosis of subclinical pre-HF (stage B HF), but are acquired for exploratory and future analysis. The acquired images are transferred to the study center for offline analysis using CVI42 version 6.3.1 (Circle Cardiovascular Imaging Inc., Calgary, Alberta, Canada). The analysis follows the current consensus recommendations for the standardized image interpretation and post-processing [16]. All personnel were trained at the CMR-Academy of the Deutsches Herzzentrum der Charité (CMR-Academy, Deutsches Herzzentrum der Charité, Berlin, Germany) and all study investigators are certified according to the Society of Cardiovascular Magnetic Resonance (SCMR) regulations. Structured reports were generated using Centricity Cardio Workflow version 7.0 (GE HealthCare Technologies Inc, Chicago, Illinois).

### 2.5 Diagnostic reporting

All participants receive a comprehensive report containing their CMR and laboratory results. This report will include information about their left ventricular GLS and other relevant findings. The report also includes recommendations for guideline-compliant pharmacotherapy, risk factor modifications, and lifestyle optimizations. In case of clinically relevant incidental findings, participants are informed and referred to appropriate specialist care.

### 2.6 Stratification and study endpoints

In accordance with the 2022 American Heart Association (AHA) guidelines for the management of HF and using previously established cutoffs of the left ventricular GLS, all participants will be categorized as healthy (GLS < −15%) or pre-HF (GLS ≥ −15%) [4], [17]. The primary endpoint of this study is the prevalence of subclinical pre-HF (stage B HF) in an urban population. Secondary endpoints include therapy adherence and the range of quality of life among individuals with subclinical pre-HF (stage B HF) in urban Germany and the prevalence of CKD. CKD is defined as an estimated glomerular filtration of < 60 mL/min at the time of the study visit.

### 2.7 Statistical considerations

The primary objective is to determine the prevalence of subclinical pre-HF (stage B HF) in an urban population. With a target sample size of 450 participants and assuming that the true prevalence is similar to that in rural areas (23%, [1023/4509], preliminary data) [10], we can determine the prevalence of subclinical pre-HF (stage B HF) with an accuracy of up to 4 percentage points (half-width of the 95% confidence interval). To accurately assess the preventive impact, we plan to analyze data from 450 participants over an additional 10-year follow-up period. To account for a potential dropout rate of about 25% (including non-analyzable CMR images due to artifacts and losses to follow-up), we aim to enroll a total of approximately 600 participants. We will perform the statistical analysis to determine the prevalence of subclinical pre-HF (stage B HF), similarly to the method used in the HERZCHECK trial [9]. Study data is collected and managed using REDCap electronic data capture tools hosted at Charité – Universitätsmedizin Berlin, Germany [18], [19].

### 2.8 Follow-up

The long-term effects of the preventive measures and therapy recommendations will be assessed during a 10-year follow-up period. To this end, patients will undergo re-evaluations at the one-, five-, and ten-year marks to assess their health status and QoL using questionnaires.

## 3 Discussion

This study describes a standardized, CMR-based approach for identifying the early, asymptomatic stages of HF in an urban population. The goal of early detection is to enable timely interventions that prevent progression to symptomatic HF, reduce mortality, and maintain patients’ quality of life.

After completing the HERZCHECK trial, which focused on rural regions and excluded urban areas, we identified the need to address this gap. The WE-CARE-HF-CMR trial is designed to provide a complete national picture. A previous study showed that the rural population is 40% more likely to suffer from clinically manifest HF than the population in big urban municipalities [11]. Therefore, it is all the more important not to draw conclusions from one setting to another.

We are applying an already tested and proven approach from the previous trial by adapting the HERZCHECK protocol to the urban population. GLS was selected as the primary screening parameter because it detects subclinical myocardial dysfunction before a decline in LVEF becomes apparent, and has demonstrated strong independent prognostic value for adverse cardiovascular outcomes [20], [21]. CMR-derived GLS can be measured quickly and reproducibly from long-axis cine images, with excellent intra- and inter-observer agreement, and remains assessable even in the presence of image artifacts [22], [23]. We acknowledge that GLS alone does not capture all components of subclinical pre-HF (stage B HF) as defined by current guidelines, particularly structural changes such as diffuse myocardial fibrosis detectable by T1 mapping. However, in a population-level screening setting using both mobile and stationary CMR platforms, cine-derived GLS offers superior cross-platform reproducibility compared to parametric mapping, which remains highly dependent on scanner hardware, field strength, and sequence implementation. The pragmatic choice of a single, well-validated functional parameter reflects the screening nature of this study. Following the original protocol ensures methodological consistency, enabling the opportunity to investigate potential differences in subclinical pre-HF (stage B HF) prevalence between urban and rural regions. Furthermore, CMR is a diagnostic method known for its high reproducibility and low operator dependency, and GLS is a robust parameter with good intra- and inter-observer variability, which helps control for confounding factors related to image acquisition and analysis [22], [23], [24].

Using mobile MRI units overseen by the Deutsches Herzzentrum der Charité (Berlin) enables large-scale, centralized quality-controlled screening. This approach was essential for providing advanced cardiac diagnostics in underserved rural regions during the HERZCHECK trial. In urban areas, while access is less constrained, the mobility of the units offers critical logistical benefits of flexibility and scalability. Thus, the WE-CARE-HF-CMR trial methodology is a feasible model for large-scale screening of subclinical HF. Additionally, deploying mobile MRI units ensures methodological comparability to the HERZCHECK trial.

The data from the HERZCHECK and the WE-CARE-HF-CMR trial together have the potential to enhance future patient care and guide the development of management strategies for this patient cohort. Additionally, the resulting large-scale quantitative database containing multimodal data (imaging, biomarkers, and structured QoL assessments) would facilitate the study of intra-individual progression of HF.

Several limitations should be acknowledged. First, the use of GLS ≥ −15% as the sole criterion for subclinical pre-HF (stage B HF) represents a simplification of the 2022 AHA/ACC definition, which encompasses a broader range of structural and functional abnormalities. This pragmatic approach was chosen to ensure methodological consistency with HERZCHECK and cross-platform reproducibility in a screening context. Second, participant recruitment through both physician referral and self-enrollment may introduce selection bias, as self-enrolling individuals in urban areas may differ systematically from the general at-risk population in terms of health awareness and socioeconomic status. Third, while the use of both mobile and stationary MRI units is a strength for logistical flexibility, potential scanner-related differences in GLS quantification should be considered, although cine-derived feature tracking is less susceptible to such variability than parametric mapping. Finally, the study is limited to five German cities and may not be generalizable to other urban populations with different demographic compositions.

## 4 Conclusion

The WE-CARE-HF-CMR study builds on the framework established by the HERZCHECK trial to deliver a comprehensive, contemporary screening strategy for the urban population. It aims to provide critical data on the prevalence of subclinical pre-HF (stage B HF) among at-risk individuals in urban settings. Furthermore, the study will assess the feasibility, patient engagement, and incremental diagnostic value of CMR as part of population-based screening programs. Its findings may support the implementation of targeted screening programs to enable early intervention and prevent the progression to symptomatic HF.

## Funding

The WE-CARE-HF-CMR trial is funded by AstraZeneca Germany, Philips Healthcare, and the German Heart Center Foundation. It is conducted under the patronage of the National Heart Alliance and the Deutsche Herzstiftung.

## Author contributions

**Jeffrey Ji-Peng Li:** Writing – original draft, Visualization, Validation, Project administration, Methodology, Investigation, Formal analysis, Data curation, Conceptualization. **Gisela Thiede:** Writing – review & editing, Visualization, Supervision, Resources, Project administration, Methodology, Funding acquisition, Conceptualization. **Collin Götze:** Writing – review & editing, Investigation, Data curation. **Vasileios Exarchos:** Writing – review & editing, Validation, Investigation, Formal analysis, Data curation. **Cyrill Meuwly:** Writing – review & editing, Investigation, Data curation. **Stefanie Maria Werhahn:** Writing – review & editing. **Rebecca Elisabeth Beyer:** Writing – review & editing. **Maximilian Leo Müller:** Writing – review & editing, Methodology, Conceptualization. **Tamar Bigvava:** Writing – review & editing. **Christian Stehning:** Writing – review & editing, Resources, Methodology. **Bjoern Andrew Remppis:** Writing – review & editing, Supervision, Methodology, Funding acquisition, Conceptualization. **Johannes Wieditz:** Writing – review & editing, Formal analysis. **Stefan Sack:** Writing – review & editing, Resources, Funding acquisition. **Tobias Heer:** Writing – review & editing, Resources, Funding acquisition. **Michael Sroka:** Writing – review & editing, Resources. **Eike Nagel:** Writing – review & editing, Supervision, Funding acquisition, Conceptualization. **Allison G. Hays:** Writing – review & editing, Conceptualization. **Grietje Duvigneau:** Writing – review & editing, Resources. **Stefan Blankenberg:** Writing – review & editing, Funding acquisition, Conceptualization. **Henning Steen:** Writing – review & editing, Supervision, Funding acquisition, Conceptualization. **Norbert Frey:** Writing – review & editing, Supervision, Funding acquisition, Conceptualization. **Stephan Baldus:** Writing – review & editing, Resources, Funding acquisition. **Katharina Seuthe:** Writing – review & editing, Resources, Funding acquisition. **Rolf Lorbach:** Writing – review & editing, Resources​​​​​​. **Florian Rödicker:** Writing – review & editing, Resources, Funding acquisition. **Sarah Scharf:** Writing – review & editing, Resources, Funding acquisition. **Ingo Hilgendorf:** Writing – review & editing, Supervision, Resources, Funding acquisition. **Ulf Landmesser:** Writing – review & editing, Supervision, Resources, Funding acquisition, Conceptualization. **Tim Friede:** Writing – review & editing, Supervision, Methodology, Formal analysis. **Volkmar Falk:** Writing – review & editing, Supervision, Resources, Funding acquisition. **Patrick Doeblin:** Writing – review & editing, Validation, Supervision, Methodology, Formal analysis, Data curation. **Sebastian Kelle:** Writing – review & editing, Writing – original draft, Visualization, Validation, Supervision, Resources, Project administration, Methodology, Investigation, Funding acquisition, Formal analysis, Data curation, Conceptualization.

## Declaration of Generative AI and AI-assisted technologies in the writing process

No generative AI was used during the preparation of this manuscript.

## Declaration of competing interests

The authors declare the following financial interests/personal relationships which may be considered as potential competing interests:Sebastian Kelle reports financial support was provided by AstraZeneca GmbH. Sebastian Kelle reports financial support was provided by Philips Healthcare. Sebastian Kelle reports financial support was provided by German Heart Center Foundation. Jeffrey Ji-Peng Li reports a relationship with Deutsche Herzstiftung that includes: funding grants. Rebecca Elisabeth Beyer reports a relationship with German Research Foundation that includes: funding grants. Christian Stehning reports a relationship with Philips Healthcare that includes: employment. Tim Friede reports a relationship with German Research Foundation that includes: funding grants. Tim Friede reports a relationship with German Ministry of Education and Research (BMBF) that includes: funding grants. Tim Friede reports a relationship with European Commission that includes: funding grants. Tim Friede reports a relationship with Actimed Therapeutics that includes: consulting or advisory. Tim Friede reports a relationship with Apellis Pharmaceuticals, Inc that includes: consulting or advisory. Tim Friede reports a relationship with ASLAN Pharmaceuticals Pte Ltd that includes: consulting or advisory. Tim Friede reports a relationship with AstraZeneca GmbH that includes: consulting or advisory. Tim Friede reports a relationship with Bayer AG that includes: consulting or advisory. Tim Friede reports a relationship with BerlinHeals that includes: consulting or advisory. Tim Friede reports a relationship with Boehringer Ingelheim GmbH that includes: consulting or advisory. Tim Friede reports a relationship with Biosense Webster Inc that includes: consulting or advisory. Tim Friede reports a relationship with Bristol-Myers Squibb Company that includes: consulting or advisory. Tim Friede reports a relationship with Cardior that includes: consulting or advisory. Tim Friede reports a relationship with CSL Behring GmbH that includes: consulting or advisory. Tim Friede reports a relationship with CVRx Inc that includes: consulting or advisory. Tim Friede reports a relationship with Daiichi Sankyo Europe GmbH that includes: consulting or advisory. Tim Friede reports a relationship with eCovery that includes: consulting or advisory. Tim Friede reports a relationship with Enanta Pharmaceuticals Inc that includes: consulting or advisory. Tim Friede reports a relationship with Fresenius Kabi Germany that includes: consulting or advisory. Tim Friede reports a relationship with Galapagos that includes: consulting or advisory. Tim Friede reports a relationship with IQVIA that includes: consulting or advisory. Tim Friede reports a relationship with Johnson & Johnson that includes: consulting or advisory. Tim Friede reports a relationship with Novartis that includes: consulting or advisory. Tim Friede reports a relationship with PINK Aktiv gegen Brustkrebs that includes: consulting or advisory. Tim Friede reports a relationship with PPD that includes: consulting or advisory. Tim Friede reports a relationship with Priothera that includes: consulting or advisory. Tim Friede reports a relationship with Recardio Inc that includes: consulting or advisory. Tim Friede reports a relationship with Recordati that includes: consulting or advisory. Tim Friede reports a relationship with Relaxera that includes: consulting or advisory. Tim Friede reports a relationship with Roche that includes: consulting or advisory. Tim Friede reports a relationship with Sarfez that includes: consulting or advisory. Tim Friede reports a relationship with UpstreamBio that includes: consulting or advisory. Tim Friede reports a relationship with Viatris Inc that includes: consulting or advisory. Tim Friede reports a relationship with VICO Therapeutics that includes: consulting or advisory. Tim Friede reports a relationship with Vifor that includes: consulting or advisory. Patrick Doeblin reports a relationship with Philips Healthcare that includes: travel reimbursement. Sebastian Kelle reports a relationship with American Heart Association Inc that includes: funding grants. Sebastian Kelle reports a relationship with Philips Healthcare that includes: funding grants. Sebastian Kelle reports a relationship with German Research Foundation that includes: funding grants. If there are other authors, they declare that they have no known competing financial interests or personal relationships that could have appeared to influence the work reported in this.
